# Colorectal Cancer Association of Canada consensus meeting: raising the standards of care for early-stage rectal cancer

**DOI:** 10.3747/co.v16i6.505

**Published:** 2009-12

**Authors:** Colorectal Cancer Association of Canada

**Keywords:** Early-stage rectal cancer, consensus statement, raising the standard for rectal cancer, multidisciplinary guidelines for early rectal cancer

## Abstract

The purpose of the meeting reported here was to develop a set of national evidence-based standards for assessing and managing patients with potentially resectable rectal cancer. This report represents the consensus of the multidisciplinary group of Canadian rectal cancer experts attending that meeting.

## 1. TERMS OF REFERENCE

### 1.1 Purpose

The purpose of the meeting reported here was to develop a set of national evidence-based standards for assessing and managing patients with potentially resectable rectal cancer. This report represents the consensus of the multidisciplinary group of Canadian rectal cancer experts attending that meeting.

### 1.2 Participants

A representative group of Canadian rectal cancer experts from the key disciplines (surgical, medical and radiation oncology, pathology, radiology) involved in managing resectable rectal cancer were invited (Table I).

### 1.3 Target Audience

- Health care professionals involved in the care of patients with potentially curable rectal cancer
- Stakeholders (provincial cancer agencies, hospitals, and so on) responsible for program and funding decisions related to the management of potentially resectable cancer
- Patient advocacy and education groups such as the Colorectal Cancer Association of Canada

### 1.4 Basis of Recommendations

All recommendations are based on a structured presentation and discussion of the best available evidence.

## 2. PREAMBLE

### 2.1 Application of Recommendations

These standards provide the basis for a discussion with patients regarding management options. Treatment plans will depend on a more complete discussion of the risks and benefits of proposed therapies with individual patients.

Significant progress has been made in improving outcomes for patients with potentially resectable rectal cancer; however, further improvement is necessary. Offering patients the option of participating in clinical trials should be a priority.

Optimally, the approach for assessing and managing patients with rectal cancer should involve a collaborative, multidisciplinary team (including all relevant medical specialties and allied health professionals). For example, optimal rectal cancer management is predicated on open communication and quality assurance between the surgeon and the pathologist describing the extent of disease of the surgical specimen for optimal postsurgical treatment choices.

Radiologic assessment and imaging should be completed within 2–3 weeks to ensure that the appropriate information is available to make timely management decisions.

## 3. QUESTIONS AND CONSENSUS STATEMENTS

### Question 1

For complete clinical staging of rectal cancer, what should the standard diagnostics and reporting be (preoperative assessment)?

### Consensus Statement 1

All diagnostics should be completed within a *timely* period (42 days to treatment, including imaging within the first 2–3 weeks), starting from the date of biopsy.

Services should include:

- Colonoscopy
- Imaging
  - Computed axial tomography of thorax, abdomen, and pelvis AND magnetic resonance imaging (mri)
  - Slices of 3–4 mm should be routine
  - Mesorectal margin measurements or circumferential resection margin (crm) with tumour distance should be reported
  - Measurements for staging criteria should be provided (see the radiology protocol in Appendix A)
- When available, endorectal ultrasonography may be complementary to mri in some T1/2 patients to better delineate T-stage

This statement utilizes Beets–Tan *et al.* 2001 1, Brown *et al.* 2003 2, Filippone *et al.* 2004 3, Nagtegaal *et al.* 2002 4, Iafrate *et al.* 2006 5, Kapiteijn *et al.* 2001 6, and Harisinghani *et al.* 2003 7.

### Question 2

For complete clinical staging of rectal cancer, what should constitute standard pathology reporting?

### Consensus Statement 2

For complete clinical staging of rectal cancer, synoptic reporting in accordance with the College of American Pathologists (cap) protocol for the examination of specimens from patients with primary carcinomas of colon and rectum, based on the American Joint Committee on Cancer and International Union Against Cancer TNM, to include these points:

- Total mesorectal excision (tme) quality
  - Macroscopic assessment of mesorectum (complete, partially complete, or incomplete)
- crm status
  - Positive if tumour is at 1 mm or less from the crm or if a lymph node with metastasis is at 1 mm or less from the crm
- If neoadjuvant therapy was received, pathologic tumour response grading should be recorded

For the complete protocol, see the cap template (Appendix B).

This statement utilizes Nagtegaal and van Krieken 2002 8, Quirke 1998 9, Heald and Ryall 1986 10, Dworak *et al.* 1997 11, Washington *et al.* 2008 13, Smith *et al.* 2008 14, Kapiteijn *et al.* 2001 15, Nagtegaal and Quirke 2008 16, Rödel *et al.* 2005 17, Glynne–Jones *et al.* 2006 18,19, Ruo *et al.* 2002 20, Nagtegaal *et al.* 2002 21, and Parfitt and Driman 2007 22.

### Question 3

Which neoadjuvant radiation protocol or protocols should be standard when combined with chemotherapy?

### Consensus Statement 3

Preoperative neoadjuvant radiotherapy is the standard of care for clinically staged ii and iii patients. Long-course radiation (minimum of 45 cGy over 5 weeks) with fluoropyrimidine chemotherapy or short-course radiation without chemotherapy can be considered. A multidisciplinary team approach (with or without a tumour board) is important to review individual cases and reach consensus on the appropriate course of treatment (short- vs. long-course radiation).

This statement utilizes Bujko *et al.* 2004 23, Marijnen *et al.* 2003 24, Swedish Rectal Cancer Trial 1997 25, Bosset *et al.* 2006 26, Gérard *et al.* 2006 27, and Sauer *et al.* 2004 28.

### Question 4

Which neoadjuvant chemotherapy protocol or protocols should be standard when combined with long-course radiation?

### Consensus Statement 4

The optimal fluoropyrimidine-based chemotherapy is based on extrapolation of data from randomized trials of combined-modality chemoradiation used in the postoperative setting. Use fluoropyrimidine-based chemotherapy with long-course radiation. Protracted fluoropyrimidine is preferable to bolus 5-fluorouracil because of improved tolerability and similar efficacy, as seen in the largest and most recent randomized trial (int 0144) 29.

This statement utilizes Smalley *et al.* 2006 29, Wong *et al.* 2008 30, and O’Connell 1994 31.

### Question 5

What should be the surgical standard of care for rectal cancer?

### Consensus Statement 5

All stage ii–iii rectal cancers should be considered for neoadjuvant treatment. For all rectal cancers undergoing radical surgery, tme principles must be followed. Surgeons treating rectal cancer patients should be familiar with the tme surgery. Quality should be assured through independent evaluations by the surgeon and the pathologist. Synoptic operative reporting is encouraged.

Trans-anal excision represents an oncologic compromise for most rectal cancer patients. Consider it only in patients with comorbidities, realizing that it requires excellent preoperative assessment and high intraoperative expertise.

Because trans-anal endoscopic microsurgery is a new approach for local excision, patients being treated using this approach should preferably be enrolled in trials or prospective follow-up studies.

This statement utilizes MacFarlane *et al.* 1993 32, Cecil *et al.* 2004 33, Dahlberg *et al.* 1998 34, Martling *et al.* 2000 35, Brown and Daniels 2005 36, Dubé *et al.* 1997 37, Karanjia *et al.* 1992 38, Ricciardi 2007 39, Murphy 2008 40, Ptok 2007 41, van den Brink 2004 42, Wibe 2002 43, Okabe 2004 44, and Nash 2009 45.

### Question 6

What criteria should be standard for handling, evaluating, and reporting on the surgical specimen?

### Consensus Statement 6

The surgeon should be aware of the standard macroscopic evaluation (grades 1, 2, 3) of the surgical specimen immediately after removal of the rectum. The pathologist receiving the specimen should also grade the macroscopic quality of the excision, independently of grading by the surgeon. Optimal management is predicated on productive, open communication between the surgeon and the pathologist so that quality assurance and appropriate mechanisms for evaluation and improvement can be achieved (see also consensus statement 5). Collaboration is mandatory for optimal evaluation; that is, margin assessment, surgical difficulty encountered, neoadjuvant treatment given to the patient must be communicated. (For optimal assessment of the specimen, the pathologist has to be informed if neoadjuvant therapy was administered.)

This statement takes account of Nagtegaal and van Krieken 2002 8, Quirke 1998 9, Dworak 1997 11, Washington *et al.* 2008 13, Smith 2008 14, Kapiteijn 2001 15, Nagtegaal and Quirke 2008 16, Nagtegaal 2002 21, and Parfitt and Driman 2007 22.

### Question 7

What is the standard adjuvant chemotherapy post neoadjuvant treatment and surgery?

### Consensus Statement 7

All patients should be considered for 4–6 months of fluoropyrimidine-based therapy. Based on extrapolation of phase III trials for adjuvant treatment of colon cancer, adjuvant oxaliplatin-based therapy should be considered for patients at high risk for recurrence, including, but not limited to those who are

- ypN-positive.
- crm-positive.

This statement utilizes Sauer *et al.* 2004 28, Wong *et al.* 2008 29, André *et al.* 2009 46, and Kuebler *et al.* 2007 47.

## 5. CONFLICTS OF INTEREST

Participants disclosed potential conflicts of interest within the past 2 years:

- Scott Berry: Advisory boards for Sanofi–Aventis
- Celia Marginean: None
- Carole Richard: None
- Andrew Smith: None
- Te Vuong: Work as a consultant for Sanofi–Aventis

The Colorectal Cancer Association of Canada and the authors acknowledge the sponsors who provided unrestricted grants: Sanofi–Aventis, Bristol–Myers Squibb, and Amgen.

## Figures and Tables

**TABLE I:** Participants in the Colorectal Cancer Association of Canada consensus meeting, December 8, 2008, Montreal, Quebec

Chairs	
Scott Berry	Medical Oncologist, Sunnybrook Health Sciences Center, Odette Cancer Center, Toronto, ON
Te Vuong	Radiation Oncologist, McGill Cancer Center, Montreal, QC
Andy Smith	Surgeon, Sunnybrook Health Sciences Center, Odette Cancer Center, Toronto, ON
Carole Richard	Surgeon, Centre hospitalier de l’Universite de Montreal, Montreal, QC
Celia Marginean	Pathologist, Ottawa Hospital, Ottawa, ON
Advisory Board members	
Shun Wong	Radiation Oncologist, Sunnybrook Health Sciences Center, Odette Cancer Center, Toronto, ON
Jean Maroun	Medical Oncologist, Ottawa Hospital Cancer Center, Ottawa, ON
Barb Melosky	Medical Oncologist, British Columbia Cancer Agency, Vancouver, BC
Thiery Alcindor	Medical Oncologist, McGill Cancer Center, Montreal, QC
Rasmy Loungnarath	Surgical Oncologist, Centre hospitalier de l’Universite de Montreal, Montreal, QC
Ralph Wong	Medical Oncologist, Cancer Care Manitoba, Winnipeg, MB
Kartik Jhavery	Radiologist, University Health Network, Princess Margaret Hospital, Toronto, ON
Sophie Lavertu	Radiation Oncologist, Centre hospitalier de l’Universite de Montreal, Montreal, QC
Erin Kennedy	Surgeon, University Health Network, Princess Margaret Hospital, Toronto, ON
Pierre Major	Medical Oncologist, Juravinski Cancer Center, Hamilton, ON
Eric Chen	Medical Oncologist, University Health Network, Princess Margaret Hospital, Toronto, ON
Audet Pascale	Radiologist, Centre hospitalier de l’Universite de Montreal, Montreal, QC
David Donath	Radiation Oncologist, Centre hospitalier de l’Universite de Montreal, Montreal, QC
Hugo Villeneuve	Radiation Oncology Resident, Centre hospitalier de l’Universite de Montreal, Montreal QC
Aaron Pollett	Pathologist, Mt. Sinai Hospital, Toronto, ON
Eugene Hsieh	Pathologist, Sunnybrook Health Sciences Center, Odette Cancer Center, Toronto, ON
Christine Cripps	Medical Oncologist, Ottawa Hospital Cancer Center, Ottawa, ON
Host	
Colorectal Cancer Association of Canada	Barry Stein, President
Meeting organizer and scribe	
Shaniah Leduc	CancerInsight
Observers	
Charles Pitts	OroAlliance
Patricia Brooks Lemoine	Colorectal Cancer Association of Canada
Manon Jobin	Amgen
Barb Burgess	Amgen
Nermine Ibrahim	Sanofi-Aventis
Therese Boucher	Sanofi-Aventis
Scott Leduc	CancerInsight

## APPENDIX A RADIOLOGY REPORTING TEMPLATE

### MAGNETIC RESONANCE IMAGING PROTOCOL

- Phased-array coil
- Field strength: 1.5 T or more
- High-resolution matrix T2 images
- Small field of view (<25 cm)
- Thin section (3–4 mm)
- Axial, coronal, and sagittal planes
- Oblique planes perpendicular to the tumour
- Gadolinium-enhanced imaging

### STANDARDIZED IMAGING REPORT

#### All Tumours

- Craniocaudal tumour extent
- Distance from anal verge
- T stage
- Circumferential (radial) margin–tumour distance
- Pelvic viscera and bones

#### Additions for Low-Rectal and Anorectal Tumours

- Distance from levator ani
- Distance from anorectal junction
- Involvement of sphincter complex
- Internal sphincter (partial or full)
- External sphincter and beyond

## APPENDIX B COLLEGE OF AMERICAN PATHOLOGISTS PATHOLOGY REPORTING TEMPLATE

***Note:*** This consensus guideline is based on College of American Pathologists (cap) guideline version 6 from early 2009. An updated cap guideline (version 7) is expected to be available at the end of 2009 and should be consulted for additional pathology reporting recommendations.

- Procedure type
  - Rectal/rectosigmoid colon (low anterior resection)
  - Abdominoperineal resection
  - Trans-anal disk excision (local excision)
  - Other
- Tumour size
- Macroscopic tumour perforation
- Macroscopic assessment of mesorectum (Note 1)
  - Complete
  - Partially complete
  - Incomplete
  - Cannot be assessed
- Histologic type
  - Adenocarcinoma
  - Mucinous adenocarcinoma
  - Signet-ring cell carcinoma
  - Small cell carcinoma
  - Squamous cell carcinoma
  - Adenosquamous carcinoma
  - Medullary carcinoma
  - Undifferentiated carcinoma
  - Other (specify)
- Histologic grade
  - Cannot be assessed
  - Low grade (well differentiated to moderately differentiated)
  - High grade (poorly differentiated to undifferentiated)
- Tumour depth of invasion (pT)
  - pTX: Cannot be assessed
  - pT0: No evidence of primary tumour
  - pTis: Carcinoma *in situ*, intraepithelial (no invasion)
  - pTis: Carcinoma *in situ*, invasion of lamina propria
  - pT1: Tumour invades submucosa
  - pT2: Tumour invades muscularis propria
  - pT3: Tumour invades through the muscularis propria into the subserosa or the nonperitonealized perirectal soft tissues
  - pT4a: Tumor penetrates the visceral peritoneum
  - pT4b: Tumor directly invades adjacent structures
- Lymph node status (pN)
  - pN0: No metastases in ____ lymph nodes
  - pN1: ____ (1–3) nodes involved of ____ (total number)
  - pN2: ____ (≥4) nodes involved of ____ (total number)
- Proximal margin
  - Cannot be assessed
  - Uninvolved by invasive carcinoma
  - Involved by invasive carcinoma
- Distal margin
  - Cannot be assessed
  - Uninvolved by invasive carcinoma
  - Involved by invasive carcinoma
- Circumferential (radial) margin (Note 2)
  - Cannot be assessed
  - Uninvolved
  - Involved by invasive carcinoma or a positive lymph node [tumour or positive lymph node present 0–1 mm from margin (or both); specify distance to margin (millimetres or centimetres)]
- Lateral margin (for noncircumferential trans-anal disk excision)
  - Cannot be assessed
  - Uninvolved by invasive carcinoma [specify distance of invasive carcinoma from closest lateral margin (millimetres or centimetres)]
  - Involved by invasive carcinoma
- Neoadjuvant therapy received
  - Yes
  - No
  - Information not available
- Tumour response to neoadjuvant treatment (Note 3)
  - Present (% of fibrosis)
  - No response identified
- Vascular (large vessel) invasion
  - Not identified
  - Present
  - Indeterminate
- Lymphatic (small vessel) invasion
  - Not identified
  - Present
  - Indeterminate
- Discontinuous extramural extension (irregular tumour nodules in pericolorectal adipose tissue without histologic evidence of residual lymph node)
  - Not identified
  - Present
  - Cannot be determined

### NOTES

#### 1. Mesorectal Envelope

The nonperitonealized surface of the fresh specimen is examined circumferentially, and the completeness of the mesorectum is scored as complete, partially complete, or incomplete 8–10. The entire specimen is scored according to the worst area.

- ***Complete:***Intact bulky mesorectum with a  smooth surface. Only minor irregularities of the mesorectal surface. No surface defects greater than 5 mm in depth. No coning towards the distal margin of the specimen. After transverse sectioning, the circumferential margin appears smooth.
- ***Nearly complete:***Moderate bulk to the me sorectum. Irregularity of the mesorectal surface with defects greater than 5 mm, but none extending to the muscularis propria. No areas of visibility of the muscularis propria except at the insertion site of the levator ani muscles.
- ***Incomplete:***Little bulk to the mesorectum.  Defects in the mesorectum down to the muscularis propria. After transverse sectioning, the circumferential margin appears very irregular.

#### 2. Circumferential (Radial) Margin

In addition to addressing the proximal and distal margins, the circumferential (radial) margin (crm) must be assessed for any segment either unencased or incompletely encased by peritoneum. The crm represents the adventitial soft tissue margin closest to the deepest penetration of tumour and is created surgically by blunt or sharp dissection of the retroperitoneal or subperitoneal aspect respectively. The serosal surface (visceral peritoneum) does not constitute a surgical margin.

The distance between the tumour and the crm should be reported. The crm is considered negative if the tumour is more than 1 mm from the inked nonperitonealized surface, but should be recorded as positive if tumour is located 1 mm or less from the nonperitonealized surface. This description includes both tumour within a lymph node and direct tumour extension; however, if crm positivity is based solely on intranodal tumour, this fact should be stated (cap protocol).

#### 3. Pathologic Tumour Response to Neoadjuvant Therapy (ypN)

The tumour response to neoadjuvant chemoradiation therapy should be recorded at least as present, recording the percentage of fibrosis in respect to residual tumour (or no response identified).

The entire scarred area of the rectum has to be blocked and scrutinized meticulously for any foci of residual tumour cells. Acellular mucin pools post neoadjuvant therapy are considered to represent a pathologic complete response. Tumour regression should be assessed only in the primary tumour; lymph node metastases should not be included in the assessment.

Several grading systems for tumour response are available 11,12. A 3-point system showed good inter-observer reproducibility 12 and may be clinically important, but it is not yet validated or regularly used in patient management and is not required for accreditation purposes for the Commission on Cancer.
